# Changing Rules and Configurations During Soccer Small-Sided and Conditioned Games. How Does It Impact Teams’ Tactical Behavior?

**DOI:** 10.3389/fpsyg.2019.01554

**Published:** 2019-07-09

**Authors:** João Cláudio Machado, João Ribeiro, Carlos Ewerton Palheta, Chellsea Alcântara, Daniel Barreira, José Guilherme, Júlio Garganta, Alcides José Scaglia

**Affiliations:** ^1^Human Performance Laboratory (LEDEHU), Faculty of Physical Education and Physiotherapy, Federal University of Amazonas, Manaus, Brazil; ^2^Faculty of Physical Education, State University of Campinas, Campinas, Brazil; ^3^Centre of Research, Education, Innovation and Intervention in Sport (CIFI2D), Faculty of Sport, University of Porto, Porto, Portugal; ^4^Sports Pedagogy Research Center (NuPPE/LAPE), Federal University of Santa Catarina, Florianópolis, Brazil; ^5^Research Group for Development of Football and Futsal, Federal University of Santa Catarina, Florianópolis, Brazil; ^6^Laboratory of Sport Pedagogy (LEPE), School of Applied Sciences (FCA), State University of Campinas, Limeira, Brazil

**Keywords:** sports pedagogy, soccer, representative learning design, task constraints, tactical behavior

## Abstract

The present study aimed to investigate how team’s tactical behavior varies within and between age categories in different Small-Sided and Conditioned Games’ configurations and conditions. Twenty non-elite youth male soccer players (U15, *n* = 10, mean age = 13.5 ± 1.2 years; U17, *n* = 10, mean age = 16.3 ± 0.5 years) were selected. Thirty-six Small-Sided and Conditioned Games (SSCG) were played in both categories, namely three Representative SSCG (R-SSCG), three Maintaining Ball Possession Games (MBPG) and three Progression to Target Games (PTG) performed for each configuration (Gk+3vs3+Gk and Gk+4vs4+Gk). Teams’ tactical behavior was analyzed based on simple and composite performance indicators, as well as through Lag Sequential Analysis. Rules manipulation and SSCG configurations influenced teams’ tactical behavior on both categories, but in different ways. Teams composed by younger players presented greater difficulties in MBPG played in smaller games configuration, while Gk+4vs4+Gk configuration can be used to enhance teams’ tactical performance of younger players in R-SSCG and MBPG conditions. Moreover, increasing rules manipulations appeared to negatively impact on teams’ exploratory behavior. Therefore, practitioners should carefully manipulate key constraints to adapt task demands to players’ age category and training session’s goals in order to enhance tactical performance.

## Introduction

During their developmental process, players are continually involved in different practice contexts that strongly influence their perceptual-cognitive skills, skill acquisition, among others (Fonseca and Garganta, 2006; Ford et al., 2009, 2012; Roca et al., 2012). In this regard, the early engagement in an unstructured practice and play-oriented activities developed by the own learners (i.e., Street Soccer) seems to enhance motor and perceptual-cognitive skills of soccer players, being understood as an important representative learning environment, since such environments stimulate player’s perceptual attunement to key information sources, also present in competitive environments (Fonseca and Garganta, 2006; Scaglia, 2011, 2014; Roca et al., 2012; Machado et al., 2019).

However, nowadays, a set of formal learning environments (e.g., soccer schools, soccer clubs, physical education classes, etc.) has emerged as the most common and appropriate places for children to learn and play soccer (Machado et al., 2017, 2019). Nevertheless, traditional teaching methods utilized in such environments are usually supported by a linear pedagogy, meaning that practitioners (i.e., coaches and/or physical education teachers) provide players and students with structured training sessions or classes characterized by the repetition of drills or rehearsals, mainly focusing on the development of technical and physical skills through application of decontextualized tasks that do not promote and stimulate the players’/students’ tactical awareness and game understanding (Ford et al., 2010; Galatti et al., 2014; Chow et al., 2016). Therefore, the beneficial and stimulating environment provided by Street Soccer have been gradually replaced by a highly rigid, structured and adult-led learning environment.

The limitations underlying the application of traditional pedagogical methods have lead researchers to propose representative learning environments (player-centered and game-based approaches) that ensure suitable practical contexts for effective learning and acquisition of skills in team sports and/or physical education (Pinder et al., 2011; Chow, 2013; Davids et al., 2013; Scaglia et al., 2013; Galatti et al., 2014). In this way, the ecological dynamics recognizes the non-linearity nature of learning, highlighting the key role of constraints manipulations in shaping the performance of players/students and teams in competitive environments (Chow, 2013; Chow et al., 2016). Furthermore, such non-linear phenomena characterizing team sport settings (e.g., the unpredictability of the game environment) justifies the need for more pedagogical and methodological principles coherent with non-linear dynamics, providing a theoretical and practical framework for understanding emergent behavior (e.g., teams’ tactical performance) (Chow et al., 2016). Therefore, Non-linear Pedagogy (NLP) encompasses a powerful paradigm for understanding human movement and for designing effective teaching, coaching and training programs in sport (Chow et al., 2013, 2016; Chow and Atencio, 2014; Serra-Olivares et al., 2016).

Thus, adopting a non-linear pedagogical approach to sports training and sports pedagogy implies practitioners to carefully manipulate specific key task constraints of performance and learning environments to better adapt game demands to players’/students’ skill levels, age category and tactical training content. Several studies have already scrutinized the influence of manipulating key task constraints (e.g., field dimensions, numerical relations, etc.) on physical and physiological, technical and tactical performance (Hill-Haas et al., 2011; Aquino et al., 2017; Ometto et al., 2018), as well as on learners’ motivation on task engagement in physical education (Roure and Pasco, 2018). However, there are few studies that sought to investigate how different task conditions and configurations (e.g., Small-Sided and Conditioned Games -SSCG) impact on teams’ performance and exploratory behavior in different age categories.

Concerning the rules manipulations (conditions), previous studies (Almeida et al., 2012; Machado et al., 2016) have highlighted that the manipulation of the amount of ball touches and passes to shooting toward the opposing goal shape different tactical behaviors. Almeida et al. (2012) observed that the manipulating of the amount of ball touches (two touches) led teams to employ a more direct attacking style, while the manipulation of the amount of passes needed to shoot at the opponent’s goal led players to perform more passes, thus stimulating the maintenance of ball possession. Lizana et al. (2015) and Machado et al. (2016) used two different SSCG conditions in an attempt to emphasize progression to the target and maintain ball possession tactical principles of play highlighted by Bayer (1994). The authors have also observed that such SSCG conditions stimulated the emergence of different attacking patterns.

Still, there is a clear lack of studies discussing how the performance of players with different ages may be influenced by the use of different SSCG conditions. Moreover, it is also important to examine how specific SSCG configurations (i.e., number of players) could promote and/or inhibit teams’ performance in such game conditions. Given the aforementioned, this study aimed to investigate how team’s tactical behavior varies within and between categories (U-15 and U-17) in different SSCG configurations (Gk+3vs3+Gk and Gk+4vs4+Gk) and conditions (rules manipulation).

## Materials and Methods

### Participants

Twenty non-elite youth male soccer players (U15, *n* = 10, mean age = 13.5 ± 1.2 years; U17, *n* = 10, mean age = 16.3 ± 0.5 years), without experience in a systematic soccer game-based training were recruited from the university’s soccer program for beginners, playing in recreational context, for participating in this study. A brief explanation of the study procedures has been priory given and only those players whose parents signed the free and informed consent, approved by the Ethics Committee for Research of the author’s university, have participated.

### Design

**FIGURE 1:**
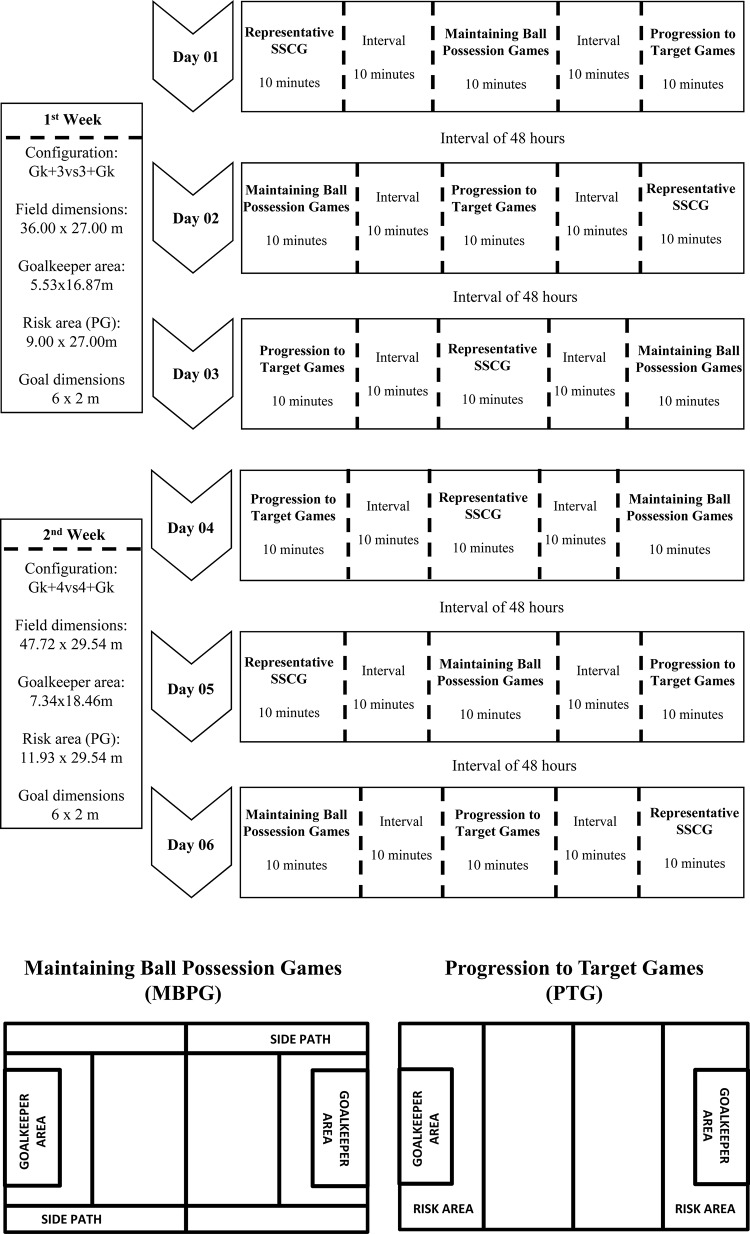
Research’s design and Small-Sided and Conditioned Games applied.

In this study, 36 Small-Sided and Conditioned Games (SSCG) were conducted, namely: three Representative Small-Sided and Conditioned Games (R-SSCG); three Maintaining Ball Possession Games (MBPG), and; three Progression to Target Games (PTG) for each games’ configuration (Gk+3vs3+Gk and Gk+4vs4+Gk) and for both age categories (U15 and U17) (see Figure 1). The reason for choosing these game configurations was based on the premise that the Gk+3vs3+Gk respects the minimum configuration that guarantees the occurrence of all tactical principles of play (Garganta, 2002; Costa et al., 2011), while the Gk+4vs4+Gk, in accordance to Garganta et al. (2013), represents the most appropriate configuration for teaching soccer since it allows a rational organization of players positioning on field, thus increasing the chances of continuously having players in all the paths and sectors of the field, facilitating the communication process established between players.

All the SSCGs were preceded by a 15-min of standardized warm-up, and two familiarization sessions were conducted for each game configuration and condition. The experimental procedure of this investigation lasted 2 weeks, aiming to respect a 48-h interval between the training sessions. In an attempt to reduce the influence of one game condition in the subsequent conditions, the order of SSCG’s was randomized. All the game configurations had 10 min of duration interspersed by 10 min of interval between them (activity/recovery ratio of 1:1) (Figure 1).

The R-SSCG followed the official rules of a soccer match, with the exception of the offside rule. This allowed us to access important information regarding the real influence of the rules manipulation in other SSCGs on teams’ tactical behavior. In all SSCG conditions, the goalkeepers (Gk) were restricted to only shot stopping activities, thus not being allowed to participate in the offensive actions.

The MBPG condition involved the manipulation of the game rules in order to emphasize the tactical principle of maintaining ball possession: (a) in the MBPG condition each player was allowed to perform a maximum of two touches on the ball, in which extra points to the opposing team were awarded for each extra touch given by the same player; (b) the players of the team with ball possession should perform constant switches of lines/zones (pre-determined in the field with cones of different colors – see Figure 1) being awarded an extra point to the team that circulate the ball from one side of the pitch to the other; (c) for each time that the team in possession of the ball achieved five consecutive passes without returning the ball to the player who have performed the last pass, the team obtained two points; and (d) the goal could only be scored after accomplishment of five consecutive passes, being the team awarded with eight points.

Finally, in the PTG condition, the rules were manipulated in order to emphasize the tactical principle of progression to the opponent’s target, in which: (a) the passes performed by the players could only be conducted toward the opponent’s goal, with backward passes being only allowed on two occasions, namely in the recovery of ball possession and in an assistance (i.e., passing to goal); (b) every goal scored preceded by a backward pass would worth five points, while a goal scored in an attacking sequence in which the last pass was performed just toward the opposing goal, would worth ten points; (c) whenever the team played into a pre-defined area delimited by the cones (risk area, i.e., area that presents risks to the opposing team – see Figure 1), would obtain three points.

### Analysis of Tactical Behavior

Tactical behavior was analyzed through: (a) Offensive Sequences Characterization System (OSCS) (Almeida et al., 2012, 2013); (b) Lag Sequential Analysis (Barreira et al., 2012, 2013).

#### Offensive Sequences Characterization System – OSCS

The OSCS, proposed by Almeida et al. (2012, 2013), was used to characterize the attacks performed by the teams during the SSCGs. The system is composed by the following performance indicators: (a) Simple indicators: duration of ball possession, number of players involved, number of ball touches, number of passes and number of shots. (b) Composite indicators: number of players involved/duration of ball possession (rhythm of collective involvement), ball touches/duration of ball possession (rhythm of ball intervention), number of passes/duration of ball possession (rate of passes), ball touches/players involved (individual contribution on ball touches), number of passes/players involved (individual contribution in ball circulation), number of passes/ball touches (attacking dynamics), and goal/shots (offensive efficacy).

#### Lag Sequential Analysis

Lag Sequential Analysis was used to analyze teams’ exploratory behaviors through the identification of the offensive patterns of play exhibited during the SSCGs. Exploratory behavior can be defined as a subsequent realization of a large amount of movement configurations under specific constraints of each performer, and the interaction with its surrounding environment (Hristovski et al., 2012; Torrents et al., 2016). Thus, LSA might be used to observe the variability of movement patterns displayed by teams and players along their offensive sequences of play. To achieve this purpose, the *SoccerEye* Observational Instrument was used (Barreira et al., 2012, 2013), which encompasses seven criteria that combine field formats with a system of categories (for more detailed information please see Table 1), namely: (a) Start of the offensive phase/ball recovery (BR); (b) Development of defense/attack transition-state (DT); (c) Progress of Ball Possession (DP); (d) End of the Offensive Phase (F); (e) Patterns of pitch space position; (f) Centre of the Game, i.e., the context of cooperation and opposition between players who actively participated and/or are able to participate in the game, in relation to the player with the ball; and (g) Spatial patterns of teams’ interaction.

**TABLE 1:** SoccerEye Observational Instrument adapted from Barreira et al. (2012).

**Criteria**	**Categories**
(1) Start of offensive phase/ball recovery (BR)	BRi: Interception; BRt: Tackle; BRgk: Intervention of the goalkeeper in the defensive phase; BRp: Defensive behavior followed by a pass; BRst: Start/restart of the offensive phase; BRv: Opponent’s violation of the laws of the game; BRc: Corner kick; BRgki: Goal kick; BRdb: Dropped ball; BRti: Throw-in
(2) Development of defense/attack transition-state (DT)	DTpsp: Positive short passing; DTnsp: Negative short passing; DTplp: Positive long passing; DTnlp: Negative Long Passing; DTpcr: Positive Crossing; DTncr: Negative Crossing; DTrb: Running with the ball; DTd: Dribbling (1x1); DTbc: Ball control; DTdu: Duel; DTs: Shooting; DTns: Opponent’s intervention with no Success; DTogk: Intervention of the goalkeeper in the offensive phase; DTdgk: Intervention of the goalkeeper in the defensive phase
(3) Progress of Ball Possession (DP)	DPpsp: Positive Short passing; DPnsp: Negative short passing; DPplp: Positive Long Passing; DPnlp: Negative Long Passing; DPpcr: Positive Crossing; DPncr: Negative Crossing; DPrb: Running with the ball; DPd: Dribbling (1x1); DPbc: Ball control: DPdu: Duel; DPs: Shooting; DPns: Opponent’s intervention with no success; DPogk: Intervention of the goalkeeper in the offensive phase; DPdgk: Intervention of the goalkeeper in the defensive phase; DPi: Violation of the laws of the game; DPc: Corner kick; DPgki: Goal kick; DPdb: Dropped Ball; DPti: Throw-in
(4) End of Offensive Phase (F)	Fws: Wide shot; Fst: Shot on target; Fso: Shot stopped, with no continuation of ball possession; Fgl: Goal; Fled: Loss of ball possession by error of the ball carrier/defender’s intervention; Fgk: Loss of ball possession by intervention of the opponent’s goalkeeper; Fo: Throwing the ball out of the pitch; Fi: Violation of the laws of the game
(5) Pattern of pitch space position	Zones 1–12
	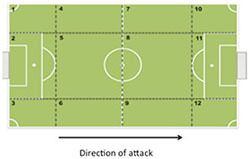
(6) Center of the Game (CJ)	Pr: Relative numerical inferiority; Pa: Absolute numerical inferiority; Pe: Pressure in numerical equality; NPe: No pressure in numerical equality; NPr: Relative numerical superiority; NPa: Absolute numerical superiority
(7) Spatial pattern of teams interaction (CEI)	EF: Ball in the empty zone (goalkeeper) versus offensive line; BF: Back line versus offensive line; BM: Back line versus mid line; BE: Back line versus exterior zone; MF: Mid line versus offensive line; MM: Mid line versus mid line; MB: Mid line versus back line; FM: Offensive line versus mid line; FB: Offensive line versus back line; EB: Exterior zone versus back line; FE: Offensive line versus empty zone (goalkeeper)

### Reliability of the Analysis

The Spearman’s Correlation Coefficient was used to evaluate data reliability regarding the performance indicators of OSCS: (i) intra-observer: the values vary between 0.83 and 0.89, with the lowest value found for the composite indicator “Ball touches/Duration,” and the highest to the simple indicator “Shots”; (ii) inter-observers: the values vary between 0.78 and 0.85, with the composite indicator “Ball touches/Duration” presenting the lowest value and the single indicator “Shots” showing the highest one. Intra and inter-observers reliability analysis was performed using SPSS 20.0 software.

Regarding the reliability of sequential analysis, two independent observers analyzed the first 45 min of the 2010 FIFA World Cup final (Spain vs. Netherlands) in two different moments, with 21 days of interval. Thus, Cohen’s Kappa Index (Cohen, 1960) was used to evaluate the intra and inter-observers reliability of SoccerEye categories. The following values were found: (i) intra-observers: 0.90 < k < 0.95; (ii) inter-observers: 0.87 < k < 0.92. SDIS-GSEQ (version 5.1, 2011) software was used to analyze data reliability.

### Statistical Analysis

*Komolgorov–Smirnov* and *Levene* tests were used to verify the normality and the homogeneity of variances, respectively, regarding the simple and composite performance indicators that compose the OSCS. Moreover, both means and standard deviations were calculated for all performance indicators. Since the multivariate normality assumption was rejected, we analyzed each independent variable independently. Therefore, multiple Mann-Whitney tests were applied to identify the main differences between categories (U15 and U17) and SSCG configurations (Gk+3vs3+Gk and Gk+4vs4+G). In addition, Kruskal–Wallis test and a *post hoc* Dunn’s test were used to compare SSCG conditions (R-SSCG, MBPG, and PTG). To calculate the magnitude of the effect of the differences, we used the formula *r* = Z/√*N* (*N* = amount of offensive sequences observed in each SSCG’s configuration and condition in both age categories), with the following values of reference: *r* = 0.2 (small magnitude); *r* = 0.5 (moderate magnitude); and *r* = 0.8 (large magnitude) (Cohen, 1988). Statistical analysis was conducted using the SPSS 20,0 software.

Also, Lag Sequential Analysis was performed through *SDIS-GSEQ* software (version 5.1, 2011), allowing to analyze teams’ exploratory behavior. This technique allows investigators to verify the existence of stability/regularity in the sequence of events above the odds that are granted by chance, using the *z*-*score* value (*z* ≥ 1.96; *p* ≤ 0.05) (Anguera, 1997). We assumed the *SoccerEye* fourth criterion — End of the Offensive Phase —, as a conduct criteria, in particular the behaviors that represented attacking efficacy, namely: (a) *wide shot* (Fws), (b) *shot on target* (Fst), (c) *shot stopped with no continuation of ball possession* (Fso), and (d) *goal* (Fgl) (see Table 1). Through a retrospective analysis of the five conducts prior to the end of the attack, the diachronic associations between the conducts were determined, in which the higher *z-value* represents the stronger associations between the events.

## Results

### Differences Between Age Categories

Table 2 shows the differences between age categories for simple and composite performance indicators. In R-SSCG, U17 teams showed a greater rhythm of ball intervention, while U15 teams presented higher individual contribution in ball circulation and attacking dynamics, showing that youngest players were more comfortable in managing task dynamics at Gk+4vs4+Gk. In MBPG, U17 teams presented longer offensive sequences, more ball touches and passes performed than U15 at Gk+3*vs*3+Gk. Furthermore, U17 teams also presented a higher rhythm of ball intervention, more individual contribution on ball touches, higher attacking dynamics and efficacy in MBPG played at Gk+3vs3+Gk configuration, indicating that older players were able to deal with high complexity levels imposed by game configuration (number of players) and condition (rules manipulated). On the other hand, U15 teams presented longer offensive sequences, more ball touches and passes performed than U17 in MBPG played at Gk+4vs4+Gk, as well as more individual contributions on ball touches and on passes performed, indicating that older players were able to perform a more direct approach to opponents’ target in those games’ configuration and condition.

**TABLE 2:** Differences between age categories in all Small-Sided and Conditioned Games.

**Small-Sided and Conditioned Games configuration**	**Performance Indicators**	**Representative SSCG**				**Maintaining Ball Possession Games**				**Progression to Target Games**			
				
		**U15**	**U17**	***p***	***r***	**U15**	**U17**	***P***	***r***	**U15**	**U17**	***p***	***R***
Gk3vs3+Gk	Duration of Ball Possession (s)	11.87 ± 9.84	13.92 ± 14.23	0.693	–0.03	17.75 ± 15.31	24.09 ± 22.28	0.050*	–0.15	12.50 ± 11.31	15.86 ± 11.6	0.014*	–0.17
	Ball Touches	7.95 ± 5.51	9.26 ± 7.40	0.274	–0.07	8.29 ± 6.45	12.36 ± 9.22	0.001*	−0.25**	8.01 ± 5.39	10.93 ± 7.39	0.005*	–0.19
	Passes	2.71 ± 1.92	3.09 ± 2.47	0.444	–0.05	5.35 ± 3.93	6.70 ± 4.87	0.037*	–0.16	2.72 ± 2.23	3.44 ± 2.85	0.007*	–0.18
	Players Involved/Duration	0.28 ± 0.17	0.29 ± 0.17	0.658	–0.03	0.27 ± 0.16	0.23 ± 0.15	0.119	–0.12	0.30 ± 0.16	0.24 ± 0.14	0.009*	–0.18
	Ball Touches/Duration	0.76 ± 0.38	0.76 ± 0.25	0.250	–0.07	0.51 ± 0.19	0.59 ± 0.18	0.002*	−0.24**	0.77 ± 0.36	0.74 ± 0.23	0.925	–0.01
	Ball Touches/Players Involved	3.17 ± 1.93	3.32 ± 1.91	0.338	–0.06	2.46 ± 1.50	3.64 ± 2.53	0.000*	−0.28**	3.06 ± 1.69	3.95 ± 2.39	0.003*	−0.20**
	Passes/Players Involved	0.99 ± 0.61	1.06 ± 0.60	0.383	–0.06	1.62 ± 0.87	1.96 ± 1.31	0.105	–0.13	0.96 ± 0.60	1.18 ± 0.52	0.001*	−0.23**
	Passes/Ball Touches	0.43 ± 0.38	0.38 ± 0.22	0.922	–0.01	0.72 ± 0.33	0.57 ± 0.16	0.001*	−0.26**	0.38 ± 0.32	0.36 ± 0.21	0.842	–0.01
	Goal/Shots	0.18 ± 0.38	0.24 ± 0.42	0.216	–0.08	0.02 ± 0.15	0.10 ± 0.30	0.042*	–0.16	0.25 ± 0.43	0.14 ± 0.34	0.041*	–0.14
Gk4vs4+Gk	Duration of Ball Possession (s)	14.20 ± 11.83	12.89 ± 10.05	0.487	–0.05	20.36 ± 15.90	17.29 ± 18.11	0.033*	–0.16	12.08 ± 10.46	14.88 ± 15.61	0.276	–0.07
	Ball Touches	8.14 ± 5.57	8.18 ± 4.94	0.584	–0.04	10.90 ± 7.75	8.29 ± 6.06	0.017*	–0.09	7.26 ± 5.00	8.68 ± 5.58	0.05*	–0.13
	Passes	3.03 ± 2.17	2.62 ± 1.84	0.228	–0.08	6.47 ± 4.52	4.87 ± 3.68	0.005*	−0.21**	2.60 ± 2.10	2.96 ± 2.31	0.294	–0.07
	Shots	0.50 ± 0.62	0.42 ± 0.53	0.461	–0.05	0.14 ± 0.39	0.13 ± 0.34	0.965	0.00	0.43 ± 0.54	0.43 ± 0.52	0.923	–0.01
	Players Involved/Duration	0.27 ± 0.16	0.30 ± 0.17	0.117	–0.10	0.28 ± 0.23	0.30 ± 0.15	0.050*	–0.15	0.28 ± 0.13	0.29 ± 0.15	0.979	0.00
	Ball Touches/Duration	0.66 ± 0.23	0.78 ± 0.38	0.011*	–0.17	0.57 ± 0.21	0.57 ± 0.22	0.551	–0.05	0.68 ± 0.26	0.77 ± 0.32	0.040*	–0.14
	Ball Touches/Players Involved	2.98 ± 1.34	3.00 ± 1.75	0.588	–0.04	2.66 ± 1.37	2.20 ± 1.05	0.012*	–0.19	2.75 ± 1.30	3.08 ± 1.63	0.127	–0.10
	Passes/Players Involved	1.03 ± 0.52	0.82 ± 0.43	0.002*	−0.20**	1.59 ± 0.78	1.28 ± 0.62	0.000*	−0.28**	0.90 ± 0.47	0.92 ± 0.56	0.787	–0.02
	Passes/Ball Touches	0.40 ± 0.22	0.36 ± 0.25	0.039*	–0.14	0.63 ± 0.17	0.60 ± 0.15	0.099	–0.13	0.40 ± 0.26	0.35 ± 0.23	0.167	–0.09

*Significant differences (*p* ≤ 0.05). ^∗∗^Small magnitude of the effect.

In PTG condition, we found that U17 teams presented longer offensive sequences, more ball touches and passes performed than U15 at Gk+3vs3+Gk configuration, as well as more individual contributions on ball touches and ball circulation. However, U15 teams presented a higher rhythm of collective involvement and higher offensive efficacy than U17 in PTG condition played at Gk+3vs3+Gk configuration, probably due to the direct style of play imposed by those teams. Regarding to PTG played at Gk+4vs4+Gk configuration, we found that U17 teams presented more ball touches and higher rhythm of ball intervention than U15.

### Differences Between Small-Sided and Conditioned Games’ Configuration

Table 3 shows the differences between SSCG configurations for simple and composite performance indicators. We found that U17 players presented a higher individual contribution to ball circulation in R-SSCG played at Gk+3vs3+Gk configuration. In MBPG, U15 teams presented more players directly involved in the offensive sequences of play, performing more touches on the ball at Gk+4vs4+Gk configuration. Furthermore, players performed more passes and presented a higher rhythm of ball intervention. However, U15 teams presented higher attacking dynamics in the Gk+3vs3+Gk configuration. The U17 teams accomplished longer offensive sequences, more ball touches, passes performed and higher offensive efficacy in MBPG played at Gk+3vs3+Gk configuration. Beyond that, players showed higher individual contributions in ball touches and ball circulation, indicating that they may have achieved the task goals through a more individual game style.

**TABLE 3:** Differences between Small-Sided and Conditioned Games configurations.

**Age category**	**Performance Indicators**	**Representative SSCG**				**Maintaining Ball Possession Games**				**Progression to Target Games**			
				
		**Gk+3vs3+Gk**	**Gk+4vs4+Gk**	***p***	***r***	**Gk+3vs3+Gk**	**Gk+4vs4+Gk**	***P***	***r***	**Gk+3vs3+Gk**	**Gk+4vs4+Gk**	***p***	***r***
U15	Players Involved	2.53 ± 0.92	2.69 ± 1.07	0.492	–0.04	3.08 ± 094	3.78 ± 1.25	0.000*	−0.32**	2.59 ± 1.03	2.59 ± 1.15	0.805	–0.02
	Ball Touches	7.95 ± 5.51	8.14 ± 5.57	0.800	–0.02	8.29 ± 6.45	10.90 ± 7.75	0.015*	–0.19	8.01 ± 5.39	7.26 ± 5.00	0.316	–0.07
	Passes	2.71 ± 1.92	3.03 ± 2.17	0.362	–0.06	5.35 ± 3.93	6.47 ± 4.52	0.050*	–0.15	2.72 ± 2.23	2.60 ± 2.10	0.783	–0.02
	Ball Touches/Duration	0.76 ± 0.38	0.66 ± 0.23	0.128	–0.10	0.51 ± 0.19	0.57 ± 0.21	0.013*	–0.19	0.77 ± 0.36	0.68 ± 0.26	0.084	–0.11
U17	Duration of Ball Possession (s)	13.92 ± 14.23	12.89 ± 10.05	0.872	–0.01	24.09 ± 22.28	17.29 ± 18.11	0.008*	−0.20**	15.86 ± 11.60	14.88 ± 15.61	0.125	–0.11
	Ball Touches	9.26 ± 7.40	8.18 ± 4.94	0.634	–0.03	12.36 ± 9.22	8.29 ± 6.06	0.001*	−0.25**	10.93 ± 7.39	8.68 ± 5.58	0.058	–0.13
	Passes	3.09 ± 2.47	2.62 ± 1.84	0.314	–0.07	6.70 ± 4.87	4.87 ± 3.68	0.004*	−0.22**	3.44 ± 2.85	2.96 ± 2.31	0.074	–0.12
	Players Involved/Duration	0.29 ± 0.17	0.30 ± 0.17	0.795	–0.02	0.23 ± 0.15	0.30 ± 0.15	0.001*	−0.24**	0.24 ± 0.14	0.29 ± 0.15	0.012*	–0.17
	Ball Touches/Players Involved	3.32 ± 1.91	3.00 ± 1.75	0.133	–0.10	3.64 ± 2.53	2.20 ± 1.05	0.000*	−0.35**	3.95 ± 2.39	3.08 ± 1.63	0.007*	–0.19
	Passes/Players Involved	1.06 ± 0.60	0.82 ± 0.43	0.004*	–0.19	1.96 ± 1.31	1.28 ± 0.62	0.000*	−0.37**	1.18 ± 0.52	0.92 ± 0.56	0.000*	–0.28^∗∗^
	Goal/Shots	0.24 ± 0.42	0.23 ± 0.42	0.867	–0.01	0.10 ± 0.30	0.02 ± 0.15	0.032*	–0.16	0.14 ± 0.34	0.24 ± 0.43	0.075	–0.12

*Significant differences (*p* ≤ 0.05). ^∗∗^Small magnitude of the effect.

Besides, we have also observed that U17 teams presented a higher rhythm of collective involvement, indicating that, in this configuration, teams were able to play more collectively, despite presenting shorter offensive sequences and less efficacy. In the PTG condition, we found that U17 teams displayed a higher rhythm of collective involvement at Gk+4vs4+Gk configuration, while U17 players showed more individual contributions on ball touches and ball circulation at Gk+3vs3+Gk configuration.

### Differences Between Small-Sided and Conditioned Games’ Conditions

Table 4 shows the differences between SSCG conditions for simple and composite performance indicators. In general, MBPG allowed teams to achieve high number of passes, with more players involved and a higher rate of passes completed. Moreover, MBPG stimulated the emergence of offensive sequences with a higher playing time comparatively to other conditions, excepting for games played by U17 teams at Gk+4vs4+Gk configurations. Also, players presented more individual contributions to passes in MBPG. However, in MBPG condition, teams performed a lower quantity of shots, as well as a lower rhythm of intervention on the ball than in other conditions, for both categories and configurations. Moreover, due to the conditions imposed by the game rules, MBPG stimulated players to perform fewer touches on the ball for each pass accomplished, in both categories and configurations.

**TABLE 4:** Differences between Small-Sided and Conditioned Games conditions.

**Age category**	**Performance Indicators**	**Gk+3vs3+Gk**			**Gk+4vs4+Gk**		
			
		**R-SSCG**	**MBPG**	**PTG**	**R-SSCG**	**MBPG**	**PTG**
U15	Duration of Ball Possession (s)	11.87 ± 9.84^#^	17.75 ± 15.31^#⁢$^	12.50 ± 11.31^$^	14.20 ± 11.83^#^	20.36 ± 15.90^#⁢$^	12.08 ± 10.46^$^
	Players Involved	2.53 ± 0.92^#^	3.08 ± 094^#⁢$^	2.59 ± 1.03^$^	2.69 ± 1.07^#^	3.78 ± 1.25^#⁢$^	2.59 ± 1.15^$^
	Ball Touches	7.95 ± 5.51	8.29 ± 6.45	8.01 ± 5.39	8.14 ± 5.57^#^	10.90 ± 7.75^#⁢$^	7.26 ± 5.00^$^
	Passes	2.71 ± 1.92^#^	5.35 ± 3.93^#⁢$^	2.72 ± 2.23^$^	3.03 ± 2.17^#^	6.47 ± 4.52^#⁢$^	2.60 ± 2.10^$^
	Shots	0.41 ± 0.53^#^	0.16 ± 0.37^#⁢$^	0.48 ± 0.57^$^	0.50 ± 0.62^#^	0.14 ± 0.39^#⁢$^	0.43 ± 0.54^$^
	Players Involved/Duration	0.28 ± 0.17	0.27 ± 0.16	0.30 ± 0.16	0.27 ± 0.16	0.28 ± 0.23	0.28 ± 0.13
	Ball Touches/Duration	0.76 ± 0.38^#^	0.51 ± 0.19^#⁢$^	0.77 ± 0.36^$^	0.66 ± 0.23^#^	0.57 ± 0.21^#⁢$^	0.68 ± 0.26^$^
	Passes/Duration	0.24 ± 0.15^#^	0.35 ± 0.16^#⁢$^	0.26 ± 0.20^$^	0.25 ± 0.17^#^	0.37 ± 0.21^#⁢$^	0.23 ± 0.15^$^
	Ball Touches/Players Involved	3.17 ± 1.93^#^	2.46 ± 1.50^#⁢$^	3.06 ± 1.69^$^	2.98 ± 1.34	2.66 ± 1.37	2.75 ± 1.30
	Passes/Players Involved	0.99 ± 0.61^#^	1.62 ± 0.87^#⁢$^	0.96 ± 0.60^$^	1.03 ± 0.52^#&^	1.59 ± 0.78^#⁢$^	0.90 ± 0.47^$&^
	Passes/Ball Touches	0.43 ± 0.38^#^	0.72 ± 0.33^#⁢$^	0.38 ± 0.32^$^	0.40 ± 0.22^#^	0.63 ± 0.17^#⁢$^	0.40 ± 0.26^$^
	Goal/Shots	0.18 ± 0.38^#^	0.02 ± 0.15^#⁢$^	0.25 ± 0.43^$^	0.17 ± 0.36^#^	0.02 ± 0.12^#⁢$^	0.20 ± 0.40^$^
U17	Duration of Ball Possession (s)	13.92 ± 14.23^#&^	24.09 ± 22.28^#⁢$^	15.86 ± 11.60^$&^	12.89 ± 10.05	17.29 ± 18.11	14.88 ± 15.61
	Players Involved	2.65 ± 0.92^#^	3.26 ± 0.82^#⁢$^	2.73 ± 1.05^$^	2.83 ± 1.16^#^	3.53 ± 1.31^#⁢$^	2.88 ± 1.29^$^
	Ball Touches	9.26 ± 7.40^#^	12.36 ± 9.22^#⁢$^	10.93 ± 7.39^$^	8.18 ± 4.94	8.29 ± 6.06	8.68 ± 5.58
	Passes	3.09 ± 2.47^#^	6.70 ± 4.87^#⁢$^	3.44 ± 2.85^$^	2.62 ± 1.84^#^	4.87 ± 3.68^#⁢$^	2.96 ± 2.31^$^
	Shots	0.53 ± 0.59^#^	0.19 ± 0.42^#⁢$^	0.44 ± 0.64^$^	0.42 ± 0.53^#^	0.13 ± 0.34^#⁢$^	0.43 ± 0.52^$^
	Players Involved/Duration	0.29 ± 0.17^#&^	0.23 ± 0.15^#^	0.24 ± 0.14^&^	0.30 ± 0.17	0.30 ± 0.15	0.29 ± 0.15
	Ball Touches/Duration	0.76 ± 0.25^#^	0.59 ± 0.18^#⁢$^	0.74 ± 0.23^$^	0.78 ± 0.38^#^	0.57 ± 0.22^#⁢$^	0.77 ± 0.32^$^
	Passes/Duration	0.26 ± 0.14^#^	0.33 ± 0.12^#⁢$^	0.25 ± 0.12^$^	0.23 ± 0.15^#^	0.33 ± 0.13^#⁢$^	0.25 ± 0.21^$^
	Ball Touches/Players Involved	3.32 ± 1.91^&^	3.64 ± 2.53	3.95 ± 2.39^&^	3.00 ± 1.75^#^	2.20 ± 1.05^#⁢$^	3.08 ± 1.63^$^
	Passes/Players Involved	1.06 ± 0.60^#&^	1.96 ± 1.31^#⁢$^	1.18 ± 0.52^$&^	0.82 ± 0.43^#^	1.28 ± 0.62^#⁢$^	0.92 ± 0.56^$^
	Passes/Ball Touches	0.38 ± 0.22^#^	0.57 ± 0.16^#⁢$^	0.36 ± 0.21^$^	0.36 ± 0.25^#^	0.60 ± 0.15^#⁢$^	0.35 ± 0.23^$^
	Goal/Shots	0.24 ± 0.42^#^	0.10 ± 0.30^#^	0.14 ± 0.34	0.23 ± 0.42^#^	0.02 ± 0.15^#⁢$^	0.24 ± 0.43^$^

*^#^Significant differences between Representative SSCG and Maintaining Ball Possession Games at the same category and SSCG’s configuration; ^$^Significant differences between Maintaining Ball Possession Games and Progression to Target Games at the same category and SSCG’s configuration; ^&^Significant differences between Representative SSCG and Progression to Target Games at the same category and SSCG’s configurations.*

Differences between R-SSCG and PTG were also observed. The U15 teams presented a higher individual contribution for ball circulation in R-SSCG played at Gk+4vs4+Gk configuration than in PTG, reinforcing the assumption that this configuration might benefit players’ participation in the game flow. In PTG played by U17 teams at Gk+3vs3+Gk configurations, we observed that such game condition stimulated an individual playing style, verified by a greater individual contribution on ball intervention than in R-SSCG played in the same configuration. Additionally, we observed that the R-SSCG promoted a higher rhythm of collective involvement than the PTG played in this same configuration (Gk+3vs3+Gk). These results might indicate that this configuration has prompted the oldest age group to play more collectively in the R-SSCG, despite being a smaller configuration.

### Analysis of Offensive Patterns of Play

In order to better understand the analysis of offensive patterns of play, we would like to emphasize that *SoccerEye* observational tool takes into account both transition and organization moments of the attack. Defense/attack transition (DT) represent the moments after a direct ball recovery, while in the offensive organization (DP) moment, teams are organized in their habitual settings, with a greater participation of the players in ball circulation (Barreira et al., 2012; Garganta et al., 2013).

**FIGURE 2:**
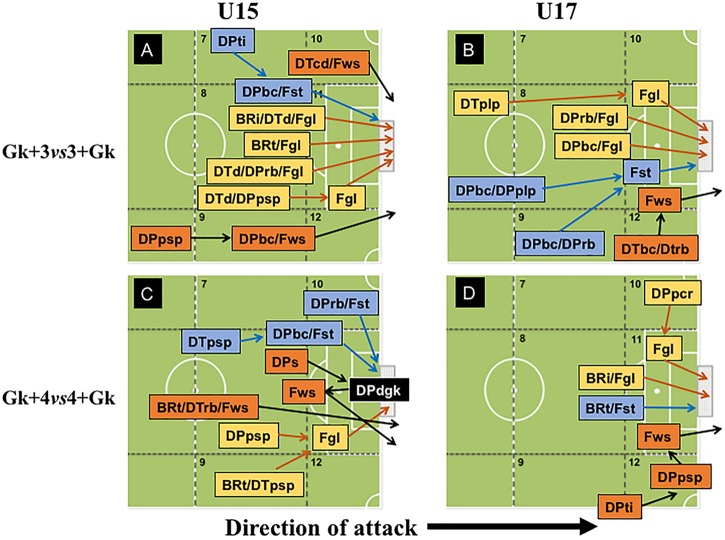
Offensive patterns of play observed in Representative SSCG at 3vs3+Gk and 4vs4+Gk configurations by U15 and U17 categories.

Regarding the R-SSCG played in the Gk+3vs3+Gk configuration (Figure 2), U15 and U17 teams presented a high variability of offensive patterns that resulted in goals scored (Fgl), as well as shots on opponents’ target (Fst) and wide shot (Fws). We can observe that the offensive patterns performed in transition, i.e., through faster attacks, were conducted after tackle (U15: *z* = 1.98), dribbling (U15: *z* = 2.77), and long pass (U17: *z* = 2.54). Moreover, goal-scoring patterns in offensive organization were also observed, where individual actions as running with the ball tended to precede goals in both U15 and U17 teams (*z* = 2.87 and *z* = 2.11, respectively). Nevertheless, we also noticed that in this configuration the U17 teams also scored goals through collective actions (DPbc: ball control – *z* = 2.40). Interestingly, the U15 teams created more goal-scoring opportunities after positive short passes, for both defense/attack transition (*z* = 3.91) and offensive organization (*z* = 5.02) in Gk+4vs4+Gk (Figure 2), showing that, in this specific configuration, teams presented more possibilities to reach the opponents’ goal through collective actions.

**FIGURE 3:**
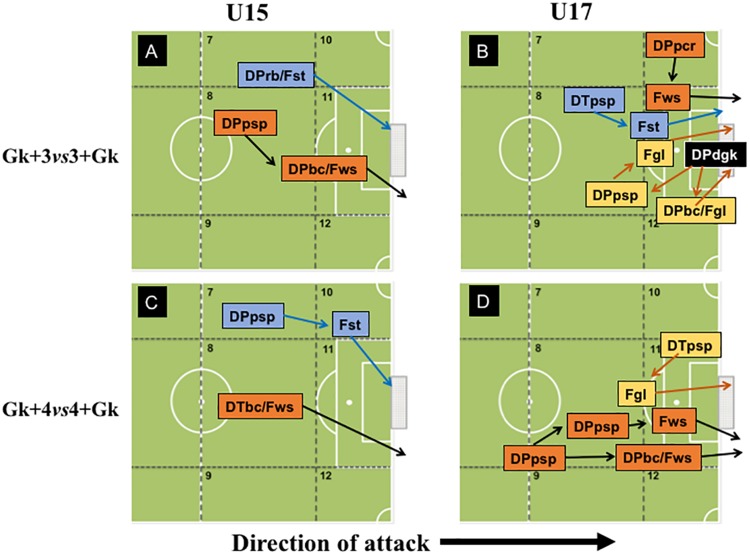
Offensive patterns of play observed in Maintaining Ball Possession Games at 3vs3+Gk and 4vs4+Gk configurations by U-15 and U-17 categories.

In the MBPG condition, teams displayed a low variability of offensive patterns of play (see Figure 3). In MBPG played in both configurations, we do not observe any offensive patterns of play that ended in goal scored for U15 teams. Notwithstanding, we verified that at Gk+3vs3+Gk configuration, shots on target (Fst) were preceded by running with the ball (DPrb – *z* = 2.58), which by the rules of the game it would not be possible, since the first rule manipulated in this condition was that each player could only perform a maximum of two touches on the ball. In the Gk+4vs4+Gk configuration, U15 teams created more shooting opportunities into opponent’s target (Fst) mainly through passes exchange (DPpsp – *z* = 6.26) in the left hand-side of the offensive midfield, exhibiting a more collective playing style.

On the other hand, in Gk+3vs3+Gk configuration, U17 teams scored goals preceded by short pass (DPpsp – *z* = 3.02) and ball control (DPbc – *z* = 4.00), both in offensive organization, showing that U17 demonstrated a more collective playing style in this SSCG configuration. Moreover, the goals scored by U17 teams in the Gk+4vs4+Gk configuration tended to occur after a positive short pass in defense/attack transition (DTpsp – *z* = 4.53), signifying that this configuration stimulated faster offensive patterns for this age category.

**FIGURE 4:**
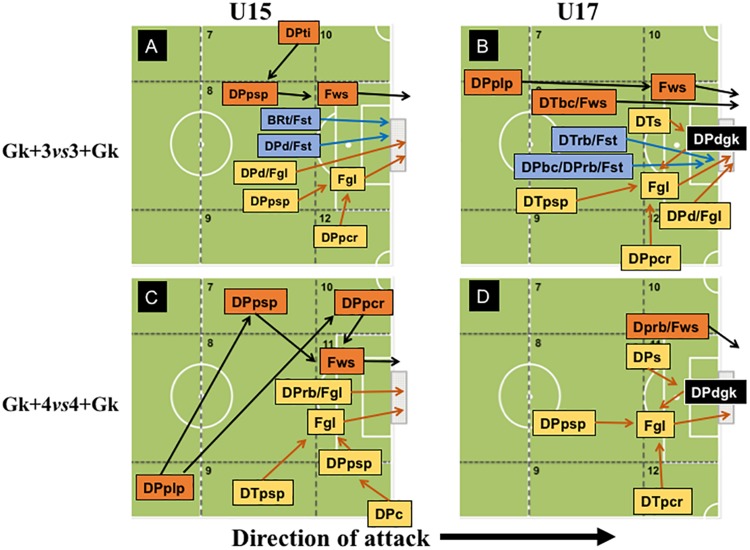
Offensive patterns observed in Progression to Target Games at 3vs3+Gk and 4vs4+Gk configurations by U-15 and U-17 categories.

Regarding PTG, it was observed that both U15 and U17 teams presented high variability of offensive pattern of play (Figure 4). In Gk+3vs3+Gk configuration, the goals scored by U15 teams were preceded by short passing (*z* = 2.36), crossing (*z* = 2.90), and dribbling (*z* = 2.03) in offensive organization, showing that in this specific SSCG’s configuration and condition, the U15 teams can be more effective (i.e., scoring goals). Alternatively, the goals scored by U17, in Gk+3vs3+Gk configuration, were usually preceded by crossing (*z* = 3.29), dribbling (*z* = 2.30) and after an intervention of the goalkeeper in the defensive phase (*z* = 3.51), all in offensive organization. However, we also observed goals scored by U17 teams through defense/attack transition, after a positive short passing (*z* = 4.80).

Moreover, in PTG played at Gk+4vs4+Gk configuration, both U15 and U17 teams scored their goals by varying their offensive patterns, either through faster attacks or implementation of a more collective playing style. The U15 teams scored their goals after a short pass (defense/attack transition: *z* = 3.02 and offensive organization: *z* = 3.05), running with the ball (*z* = 2.63) and opponent’s intervention with no success (*z* = 3.41) in offensive organization. Regarding the offensive patterns of play that ended in goals in U17 teams, for the Gk+4vs4+Gk configuration, goals tended to be preceded by a positive crossing in defense/attack transition (*z* = 2.60) or a positive short pass (*z* = 2.63), as well as by a goalkeeper’s intervention (*z* = 2.45) in offensive organization.

## Discussion

The purpose of this study was to investigate how teams’ tactical behavior varies with age (U15 and U17) in different SSCGs configurations (Gk+3vs3+Gk and Gk+4vs4+Gk) and game conditions (Representative SSCG, Maintaining Ball Possession Games, and Progression to Target Games). Based on the results, we highlight that practitioners should consider players’ age, task difficulty in which regards the application of different SSCG’s configurations, and tactical training content to design representative training tasks to enhance tactical learning.

Thus, in order to better explain the practical implications of the present study, we organized the discussion into subsections: (a) the performance of U15 and U17 teams according to the different SSCG’s conditions and configurations; (b) the rules as key task constraints that can either induce and/or stabilize specific offensive patterns of play, as well as limit players’ and teams’ exploratory behavior.

### U15 and U17 Teams’ Performance in Different SSCG’s Configurations and Conditions

Results from this study highlighted that U15 teams presented better performances in MBPG played at Gk+4vs4+Gk than in Gk+3vs3+Gk, thus completing more passes, ball touches, and displaying a higher rhythm of ball intervention. Considering the operational tactical principles defined by Bayer (1994), Serra-Olivares et al. (2016) observed that players presented greater difficulties in playing the Maintaining Ball Possession SSCGs comparatively with the Progression to Target SSCGs. Despite using goalkeepers in all SSCGs, and have investigated older age categories (U15 and U17), our results are similar to Serra-Olivares et al. (2016), which have concluded that younger players exhibited better passing performances and higher amount of ball touches in offensive sequences of play at the Gk+4vs4+Gk configuration. Moreover, this configuration provided younger players with the possibility of playing collectively, evidenced in increased players’ involvement in offensive sequences. In this perspective Aguiar et al. (2015) observed that in 4-a-side and 5-a-side SSCG formats, teams presented a higher positional occupation on field comparatively with 2-a-side and 3-a-side formats, thus suggesting that both 4-and 5-a-side configurations stimulated a more collective play style.

Additionally, U15 teams displayed better attacking effectiveness (Goal/Shots composite indicator) in PTG at Gk+3vs3+Gk than U17 teams in the same configuration. Beyond that, it is important to highlight that PTG rules stimulated attacks with shorter duration, fewer passes performed and players involved, when compared to other conditions (R-SSCG and MBPG). Also, we observed that U17 teams exhibited a higher rhythm of collective involvement at Gk+4vs4+Gk configuration and more individual contributions on ball touches and ball circulation at Gk+3vs3+Gk configuration. Thus, PTG played at Gk+3vs3+Gk configuration induced a more direct playing style, where teams tried to move forward quickly attempting to create goal-scoring opportunities through individual actions. These results are in line with those found by Folgado et al. (2012), where the authors verified that in Gk+3vs3+Gk configurations, teams composed by younger players showed a greater dispersion across the field length in relation to width, as well as an approximation between the centroids of both teams, suggesting a closer approach to the ball, which may have hampered ball-passing actions.

Information derived from the composite performance indicators allowed us to verify that U17 players presented a higher contribution to the passes exchanged in PTG played at the Gk+3vs3+Gk configuration, while U15 players participated more actively in the passes exchange in R-SSCG and MBPG played in the Gk+4vs4+Gk configuration than U17’s. This body of knowledge may suggest that U15 players and teams were more comfortable in performing ball-passing actions in R-SSCG and MBPG played at Gk+4vs4+Gk, probably because this configuration enabled a more balanced distribution of player’s on field, thus corroborating Garganta et al. (2013) and Aguiar et al. (2015) statements.

Regarding teams’ exploratory behavior, we verified that U15 teams presented a higher variability of offensive sequences that ended in a goal scored in R-SSCG played at Gk+3vs3+Gk configuration. Torrents et al. (2016) observed that players tended to explore multiple actions in tasks with higher difficulty levels (i.e., numerical disadvantage). We also observed that shots were preceded by “Running with the ball” (DPrb) in MBPG played at Gk+3vs3+Gk configuration for U15 teams, constituting a violation of the game rules, suggesting the difficulties that younger players may have faced when playing in smaller game configurations.

Therefore, we highlight that players’ age and game configuration have a significant effect on teams’ and players’ tactical performance. However, further researches are needed to carefully examine players’ skill levels within each category, to better understand their real impact on teams’ and players’ performance. Arguably, such insights will provide practitioners with important information to better organize their training sessions and/or physical education classes, and to design representative training tasks, in attempts to create an effective learning environment based on a learner-environment-centered approach.

Our results also have an important practical implication for teaching in physical education classes. Student-centered models (e.g., Sports Education, Teaching Games for Understanding, Invasion Games Competence Model) have been increasingly used to stimulate the learners’ personal and social development, also impacting on performance enhancement and learning (Farias et al., 2015). Teaching Games for Understanding (TGfU), for example, promote the learning of common principles of play in team sport games by providing students with opportunities for solving different tactical problems in different game formats, such as the principles used in this study (maintaining ball possession and progression to target) (Bunker and Thorpe, 1982; Farias et al., 2015). In this sense, the Gk+4vs4+Gk configuration, for example, might be used during learners’ first contact with soccer to emphasize the tactical problem of maintaining ball possession, without compromising the performance of the younger players/students, since smaller configurations seem to present greater difficulty to players frequently exchange passes.

### The Rules as Key Task Constraints

The game rules can be understood as important informational variables that can highly constrain players and teams behaviors (e.g., tactical patterns of a team). Regarding the differences between SSCG conditions, the MBPG games promoted longer attacking sequences, with greater number of players involved, higher amount of passes performed, and greater dynamics in ball-passing exchange. Here, players participated more actively in passing sequences, intervening less on the ball, regardless of the category and the configuration involved. Therefore, we can highlight that MBPG emphasized the operational principle of maintaining ball possession, stimulating a positional attack, hence corroborating with the findings of Lizana et al. (2015) and Machado et al. (2016).

On the other hand, we verified that U15 and U17 teams showed low variability of their attacking patterns of play in the MBPG in both configurations (Gk+3vs3+Gk and Gk+4vs4+Gk). Possibly, the use of an excessive number of game rules, which may have restricted the number of players’ possibilities for action, may explain these results. Moreover, regarding the analysis of the attacking efficacy, we observed that U15 and U17 teams in both configurations (Gk+3vs3+Gk and Gk+4vs4+Gk) presented significantly lower scores compared to other conditions (i.e., R-SSCG and PTG).

Our results corroborates the findings of Serra-Olivares et al. (2016), i.e., U15 teams displayed a higher difficulty to play the MBPGs in the Gk+3vs3+Gk configuration, since the attacks that ended in shots on target (Fst) tended to be preceded by running with the ball (DPrb), which according to the definitions of *SoccerEye* observation tool, occurs when a player gives three or more touches on the ball in progression over the field (Barreira et al., 2012). Therefore, because in the MBPG players were only allowed to give a maximum of two touches on the ball, we verified that the U15 players did not complied with the rules proposed by this game in this specific configuration (Gk+3vs3+Gk).

Moreover, by applying MBPG to a SSCG with a small configuration, i.e., Gk+3vs3+Gk, teams composed by younger players showed greater difficulties in finding functional patterns of coordination that enable them to achieve task goals, since it was not possible to identify the offensive patterns of play that resulted in goals scored. Following this logic, Davids et al. (2001) and Headrick et al. (2015) highlighted that systems that present unstable states of organization (i.e., tactically unbalanced teams) are more likely to be influenced by the manipulation of informational variables (such as game rules). Therefore, the manipulation of game rules in training sessions that intend to emphasize a specific tactical principle of play need to be carefully rethought because of the SSCG configuration that is being used, and, of course, the players’ skill level and age. In line with this perspective, Headrick et al. (2015) highlighted that through practice players may become more resistant to the external disturbances caused by the manipulation of these informational variables. Hence, this may explain the fact that U17 teams presented slightly higher variability in attacking patterns of play regarding the MBPG played at Gk+3vs3+Gk configuration, identified by the patterns that have resulted in goals scored.

Interestingly, Torrents et al. (2016) found results that are in line with our study. In fact, they highlighted that exaggerated constraints manipulation possibly hampers the attainment of specific task goals and also restricts teams’ and players’ exploratory behavior, i.e., by reducing the realization of movement configurations, since the emergence of such coordination patterns is highly dependent on the affordances perceived by players and students from their surrounding environments (Hristovski et al., 2012; Torrents et al., 2016). Therefore, training tasks that have an excessive amount of manipulated rules may limit players’ possibilities for acting in dynamical performance environments (MBPG in this study), influencing their abilities to use specified information compatible with the accomplishment of specific tactical patterns of play, hence inhibiting exploratory behavior of players and teams to occur.

Our findings underline the importance of practitioners in manipulating these informational variables in order to design better representative tasks, i.e., that are more appropriate and adequate for players’ skill level, age category and training session’s goals. We claim that manipulating key constraints in tasks used with younger players/students, or even with players/students with low skill levels, need to stimulate players’/students’ and teams’ exploratory behavior, in an attempt to increase learners’ creativity, tactical awareness and game understanding, rather than just direct their actions to templates considered as the best and unique solutions for specific game problems.

## Conclusion

The current study highlighted that U15 and U17 teams’ tactical behaviors seem to be influenced by SSCG configurations (Gk+3vs3+Gk and Gk+4vs4+Gk) and conditions (R-SSCG, MBPG, and PTG), however, in different ways: (a) teams composed by younger players presented greater difficulties in maintaining ball possession in games played in a smaller SSCG configuration; (b) Gk+4vs4+Gk configuration promoted a better tactical performance in the MBPG condition in teams composed by younger players; (c) Gk+3vs3+Gk configuration promoted a better offensive efficacy in PTG condition in U15 teams; (d) MBPG condition stimulated longer attacking sequences of play, with greater amount of passes performed, more players involved and higher participation of players in ball-passing exchanges; (e) the exaggerated amount of game rules manipulated in MBPG inhibited the variability of offensive patterns of play in both SSCG configurations and categories, and the U15 teams showed a greater difficulty in dealing with these rules in the Gk+3vs3+Gk configuration.

These results may furnish important information for practitioners, whether in clubs, sports initiation programs or in the school physical education contexts, such as: (a) the excessive manipulation of SSCG rules may be harmful to teams composed by younger players, inhibiting players and teams’ capacity for exploring different functional motor solutions or offensive patterns; (b) the Gk+4vs4+Gk configuration can be used to enhance teams’ tactical performance of younger players in R-SSCG and MBPG conditions; (c) the Gk+3vs3+Gk configuration can be used to enhance offensive efficacy of younger players in PTG condition; (d) Gk+4vs4+Gk configuration facilitates the game flow, allowing teams composed of younger players to exchange passes more frequently, maintaining ball possession, in SSCG with higher difficulty levels.

Therefore, it is important to highlight that practitioners need to carefully manipulate key task constraints (rules, number of players, pitch dimension, etc.), always considering players’/students’ age and skill levels, as well as tactical principles of play/objectives that coaches and/or teachers intend to emphasize and develop whether in training sessions or in schools.

## Data Availability

The datasets generated for this study are available on request to the corresponding author.

## Ethics Statement

This study was approved by the Ethics Committee for Research of the State University of Campinas (UNICAMP). Number: 2.250.881 (CAEE - 73222617.0.0000.5404).

## Author Contributions

JM outlined this study, collected and analyzed the data, as well as wrote and reviewed the final version of this manuscript. CP and CA collected and analyzed the data, as well as reviewed the final version of this manuscript. DB and JR technically supported the procedures regarding Lag Sequential Analysis and Performance Indicators analysis, respectively. JM and JR were responsible for statistical procedures. JGui and JGar were responsible for data interpretation and review of this manuscript. AS outlined this study, analyzed the data, and reviewed the final version of this manuscript.

## Conflict of Interest Statement

The authors declare that the research was conducted in the absence of any commercial or financial relationships that could be construed as a potential conflict of interest.
